# Book Review: Computing for Biologists: Python Programming and Principles

**DOI:** 10.3389/fgene.2016.00086

**Published:** 2016-05-11

**Authors:** Alberto Marin-Sanguino

**Affiliations:** Specialty Division for Systems Biotechnology, Faculty for Mechanical Engineering, Technische Universität MünchenMünchen, Germany

**Keywords:** python, computational biology, systems biology, programming languages, computing

Fifteen years ago a student could go through the whole curriculum in any of the life sciences without ever switching a computer on but, as bioinformatics and systems biology gained weight in the field, biologists became first users and then developers of increasingly sophisticated computer programs. Even for professionals far away from these disciplines, it is nowadays unthinkable to plan an experiment without first checking some databases, designing some primers or analyzing a sequence. These changes have created a need to include basic courses in the biology curriculum where students with little or no inclination toward computer science have to learn the ropes of programming. The main challenge when teaching such a course is to find a good balance between presenting examples that are complex enough to motivate the students and simple enough to be accessible for them. This book is precisely a guide for such an introductory course. Using python as a first programming language and assuming no previous knowledge, the authors follow a practical approach to teach the basic programming skills.

The book is structured in four parts, preceded by a twenty pages tutorial on the basics of python. The examples in each of the first three parts revolve around a unifying biological theme, which turns each of them into a simple yet interesting project. Abstract computational concepts like recursion or memoization are introduced as they are needed to solve diverse problems. At the end of each part, a problem is formulated to solve a real case study using the material covered so far. Abundant examples, additional explanations on these problems and source code with the solutions are available through he companion website. The first part starts with simple tasks like computing GC content of a DNA sequence or converting DNA to its corresponding mRNA. Through these simple examples, the usage of different data types is introduced as well as the basics of flow control and functions. The rest of the first part is dedicated to the consolidate knowledge on these basic concepts understanding the general organization of a program. These concepts are then used to find ORFs in a genome and sequences that are associated with pathogenicity in *Salmonella*. The second part covers sequence alignments, building up toward finding homology between genes and then chromosomes. It is all exemplified by comparing X chromosome in humans and Z chromosome in chicken. The third part covers phylogenetic trees and ends with mitocondrial DNA comparison between humans and neanderthals. finally, a more heterogeneous part closes the book presenting three different examples that depart from the rest: RNA folding, finding gene regulation networks and genetic algorithms.

The text is extremely well written, with clear explanations and interesting examples. The pace is slow but entertaining, ensuring that the student can keep up with the step by step explanations while sitting in front of the computer and trying things right away. No previous knowledge is assumed, and the most basic programming concepts are explained from scratch, stopping to indicate possible pitfalls and preparing the reader for potential difficulties. There are many introductory texts to Python, including some aimed to biologists. What makes this book different is that it does not focus on teaching a particular programming language or some useful algorithms. The authors present biological problems and keep the attention focused on them. Python is just taken as a vehicle to introduce very abstract concepts by example. This makes the book valuable as a guide for an undergraduate course. Even if the students never use python after the course is over, they will have acquired the basic programming skills they may need. I haven't found a book that follows this approach so successfully since “Beginning Perl for Bioinformatics” by James Tisdall, 15 years ago.

## Author contributions

The author confirms being the sole contributor of this work and approved it for publication.

## Funding

This work was funded by the German Ministry of Education and Research (BMBF) through the e:Bio initiative (project OpHeLia—0316197).

### Conflict of interest statement

The author declares that the research was conducted in the absence of any commercial or financial relationships that could be construed as a potential conflict of interest.

